# Stoichiometric Analysis of Shifting in Subcellular Compartmentalization of HSP70 within Ischemic Penumbra

**DOI:** 10.3390/molecules26123578

**Published:** 2021-06-11

**Authors:** Federica Mastroiacovo, Francesca Biagioni, Paola Lenzi, Larisa Ryskalin, Stefano Puglisi-Allegra, Ferdinando Nicoletti, Alessandro Frati, Francesco Fornai

**Affiliations:** 1 I.R.C.C.S. Neuromed, Via Atinense 18, 86077 Pozzilli, IS, Italy; federica.mast@neuromed.it (F.M.); francesca.biagioni@neuromed.it (F.B.); stefano.puglisiallegra@neuromed.it (S.P.-A.); ferdinandonicoletti@hotmail.com (F.N.); alessandro.frati@uniroma1.it (A.F.); 2 Department of Translational Research and New Technologies in Medicine and Surgery, University of Pisa, Via Roma 55, 56126 Pisa, Italy; paola.lenzi@unipi.it (P.L.); larisa.ryskalin@unipi.it (L.R.); 3 Department of Physiology and Pharmacology “V. Erspamer”, University Sapienza of Rome, 00185 Rome, Italy; 4 Neurosurgery Division, Human Neurosciences Department, Sapienza University, 00135 Rome, Italy

**Keywords:** mitochondria, autophagy-related vacuoles, chaperones, area penumbra, brain ischemia, transmission electron microscopy, quantitative morphometry, stoichiometric ultrastructural molecule detection

## Abstract

The heat shock protein (HSP) 70 is considered the main hallmark in preclinical studies to stain the peri-infarct region defined area penumbra in preclinical models of brain ischemia. This protein is also considered as a potential disease modifier, which may improve the outcome of ischemic damage. In fact, the molecule HSP70 acts as a chaperonine being able to impact at several level the homeostasis of neurons. Despite being used routinely to stain area penumbra in light microscopy, the subcellular placement of this protein within area penumbra neurons, to our knowledge, remains undefined. This is key mostly when considering studies aimed at deciphering the functional role of this protein as a determinant of neuronal survival. The general subcellular placement of HSP70 was grossly reported in studies using confocal microscopy, although no direct visualization of this molecule at electron microscopy was carried out. The present study aims to provide a direct evidence of HSP70 within various subcellular compartments. In detail, by using ultrastructural morphometry to quantify HSP70 stoichiometrically detected by immuno-gold within specific organelles we could compare the compartmentalization of the molecule within area penumbra compared with control brain areas. The study indicates that two cell compartments in control conditions own a high density of HSP70, cytosolic vacuoles and mitochondria. In these organelles, HSP70 is present in amount exceeding several-fold the presence in the cytosol. Remarkably, within area penumbra a loss of such a specific polarization is documented. This leads to the depletion of HSP70 from mitochondria and mostly cell vacuoles. Such an effect is expected to lead to significant variations in the ability of HSP70 to exert its physiological roles. The present findings, beyond defining the neuronal compartmentalization of HSP70 within area penumbra may lead to a better comprehension of its beneficial/detrimental role in promoting neuronal survival.

## 1. Introduction

When combining experimental and clinical/instrumental studies, it appears that, a surrounding (peri-infarct) area exists around a brain infarct where cells are still alive, though being on the edge of a dual fate, undergoing cell death or being rescued. Such a peri-infarct border of potentially salvageable brain matter undergoes progressive biochemical and cellular changes, each one with a specific time course [1], which develop along with maturation phenomena surrounding the core of cerebral ischemia. The specific borderline brain area under ischemic stress owing a specific metabolic demand and the timing of physiological metabolic needs may alter the fate of these cells and their final outcome. Nonetheless, occurrence of specific time-dependent compensatory changes indicates that some biochemical pathways may be induced in order to improve neuronal rescue in this borderline region. The identification of such an area in vivo in human patients is allowed by imaging procedures such as magnetic resonance spectroscopy, diffusion- and perfusion-MRI as well as positron emission tomography. The imaging provided by MRI shows a “shadow-like” intensity, which according to a Latin word, led to a definition of “area penumbra”. Imaging of focal inflammation and the amount of anaerobic glycolysis which develop within area penumbra, despite identifying the area, do not provide a marker to predict the outcome of penumbra-recruited cells. In fact, the intimate biochemical significance of area penumbra corresponds to the brain tissue surrounding the ischemic core where suppressed protein synthesis but preserved ATP is measured [2]. Such an area is routinely identified at light microscopy by a brain strip where HSP70 stress protein is induced/increased [2]. The first definition of area penumbra as a HSP70-positive peri-infarct region was provided by Kinouchi et al. [3] who proposed that such a biochemical marker has inherent prognostic significance. In fact, a number of studies indicate that HSP70 may represent a marker to predict the outcome of penumbra recruited cells. It is hypothesized that HSP70 sustains a compensatory mechanism to counteract deleterious effects of ischemia [4]. This is substantiated by a number of experimental approaches where genetic or pharmacological modulation of HSP70 expression leads to enlarge or reduce the amount of the final infarct area [5,6,7,8,9]. Thus, despite being the gold-standard biochemical marker to define area penumbra, HSP70 owns a robust significance in the pathophysiology of maturation phenomena following brain ischemia. Despite the bulk of data showing the functional effects produced by HSP70 and studies using HSP70 staining as a morphological guide to identify area penumbra at light microscopy, to our knowledge no study so far investigated the subcellular compartmentalization of HSP70 within area penumbra and how this is modified in specific conditions. For such a reason, even when considering the constitutive form of HSP70, no study so far has analyzed the functional compartmentalization of HSP70 within area penumbra compared with non-ischemic cells. Thus, when considering HSP70 as a dynamic marker of penumbra cells, which is supposed to interact beneficially with the maturation of these cells towards a rescue pathway, it is mandatory to get an insight on subcellular compartmentalization of this protein. Surprisingly, despite most experimental studies are based on light microscopy staining, as well as biochemistry and molecular biology of HSP70 within area penumbra, to our knowledge no study so far analyzed subcellular placement of HSP70 and whether this shifts within penumbra cells. Thus, in the present study we investigated the placement of HSP70 within the cell by using ultrastructural morphometry. In detail, following light microscopy with thionine staining we could grossly identify the ischemic regions and the small are to be dissected for ultrastructural studies. A specific immunostaining allowed to select the penumbra peri-infarct regions by highly selective inducible HSP70 immunostaining. Based on this topography we punched out specimen for semi-thin sections in order to select the specific area to be analyzed under quantitative morphometry using immuno-gold transmission electron microscopy to quantify the subcellular placement and trafficking of HSP70. In this way, the stoichiometric amount of constitutive HSP70 within various cell compartments from control and penumbra cells was quantified. Therefore, the specific cell compartments hosting HSP70 in baseline conditions were identified and the amount of the protein was counted along with its misplacement, which takes place in the peri-infarct area. Counts profited from stoichiometry bound immuno-gold particles. The study was carried out in the mouse brain by using a permanent model of brain ischemia induced by occlusion of the distal part of the middle cerebral artery (MCA) through cauterization of the blood vessel.

## 2. Results

### 2.1. The Outcome of Permanent Ischemia

**Figure 1:**
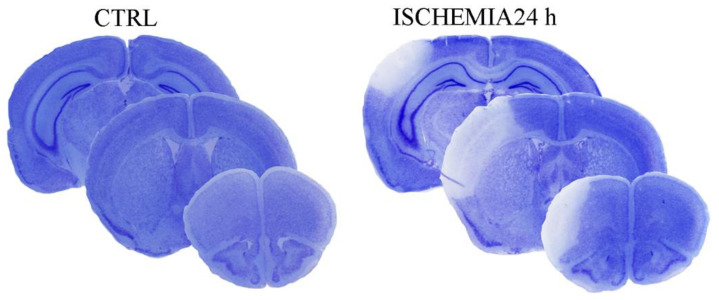
Representative thionine staining of the area penumbra. Representative slices stained with thionine from control (**left panel**) and ischemic mice (**right panel**). The extent of the ischemic lesion is shown in ischemic mice and the infarct volume was calculated from *n* = 4 mice.

In Figure 1 the outcome following the induction of permanent ischemia following MCA occlusion in C57Black mice is reported. This corresponds to a region where ischemic infarct is detectable as early as 2–3 h after MCA occlusion, and reaches its maximal size at 24 h. The extent of the infarct volume, evaluated by Nissl staining at 24 h after MCA occlusion corresponds to a rostro-caudal size of +2.34 from bregma back to −3.28 from bregma and a dorsoventral extent which depends on the rostro-caudal level and reaching the highest depth in the middle of the rostro-caudal extent, where it involves a half of the dorsoventral brain length (as shown in representative Figure 1). When measured by using a stereological procedure the brain ischemic volume corresponded to 6 + 1.5 mm^3^. The area extends from the primary motor cortex (rostral limit) to the secondary visual cortex (caudal limit) including secondary as well as primary auditory cortex.

### 2.2. The Selection of the Peri-Infarct Regions

**Figure 2:**
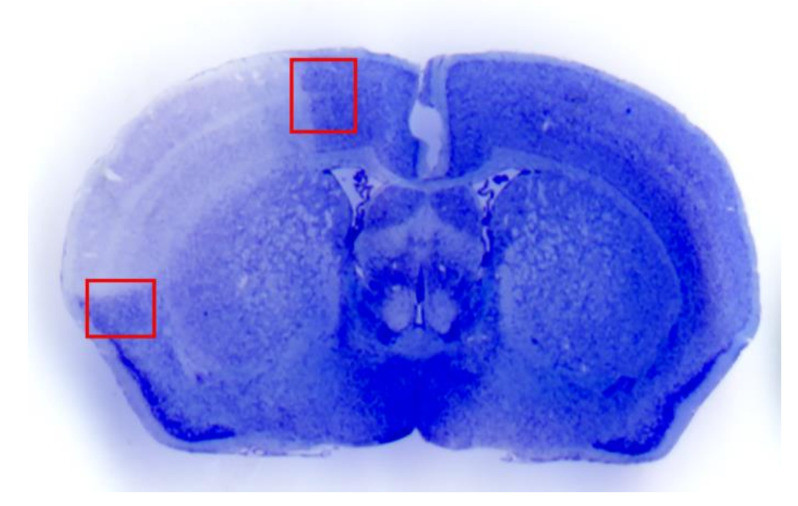
Representative picture of dissected regions. Selective brain regions of dissected area penumbra are shows in representative picture.

As reported in Figure 2, within this widespread region we aimed at two specific cortical regions where a peri-infarct area can be visualized. As shown in representative Figure 2 such an area is evident by the quite short chromatic switch from the pale white frankly necrotic region to the blue normal Nissl-stained brain matter. This is evident in Figure 2 at cortical level by red squares, which were selected both in the dorsal and the ventral extent of the peri-infarct area. These peri-infarct cortical regions were selected to study area penumbra on coronal slices in the dorsal and ventral border of the central rostro-caudal extent (selected regions from 1.94 from bregma back to 0.14 from bregma). These cortical regions correspond dorsally to the central layers of the iso-cortical primary motor cortical region, while ventrally the red square delimits a cortical region just above the limbic allocortex which defines the granular and agranular insular cortex according to the atlas of Franklin and Paxinos [10]. The HSP70 placement study proceeded in both the dorsal and ventral region both concerning immunohistochemical placement and the ultrastructural morphometry for HSP70 compartmentalization. This was done to intrinsically validate the data independently by the nature of the cortical region under analysis. In fact, the structure and physiology of the primary motor cortex markedly differ from the insular cortex which continues up the piriform allocortex and may be considered a peripheral region of the limbic system.

### 2.3. Immunohistochemistry at Light Microscopy for HSP70

**Figure 3:**
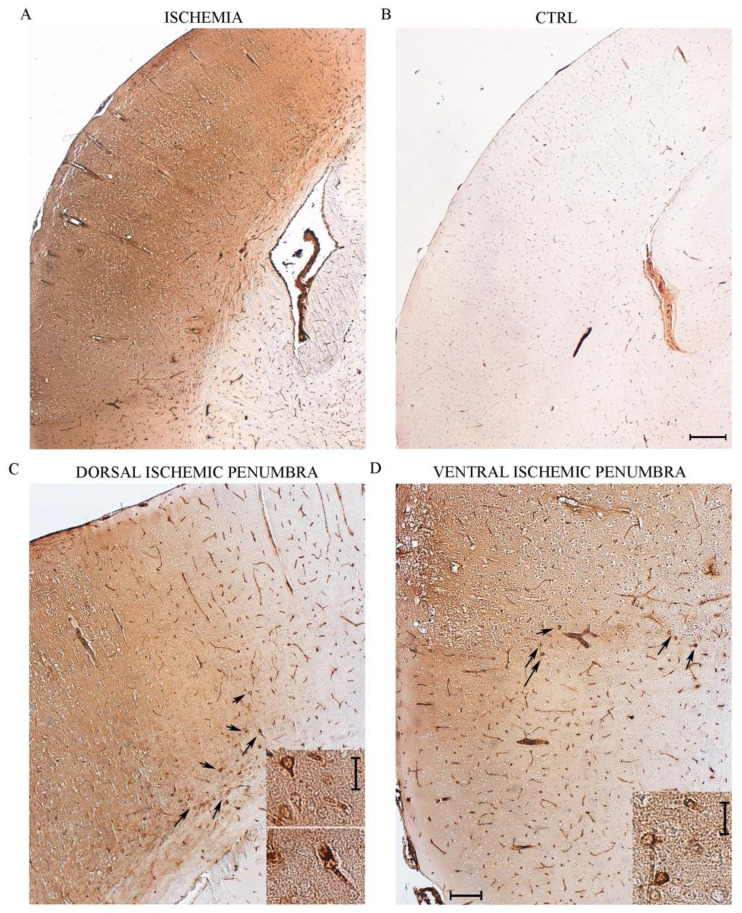
Representative HSP70-immuno-staining in the area penumbra. This picture shows Heat Shock Protein70 positive neurons in the area penumbra of ischemic (**A**) vs. control mice (**B**). In (**C**,**D**) representative pictures shows a higher magnification of dorsal and ventral ischemic area penumbra. Scale bar = 250 μm (**A**,**B**). Scale bar = 100 μm (**C**,**D**), 25 μm (**inserts**).

When immunoperoxidase treatment was carried out in the red squared regions of Figure 2, a remarkable strip of HSP70 positive neurons was evident both dorsally and ventrally in the peri-infarct areas compared with neighboring cortical tissue in the ischemic side (Figure 3A). Such a staining pattern markedly contrasts with the clean white signal being detected in the corresponding regions of a control mouse (Figure 3B). At progressive magnification the dorsal (Figure 3C) and ventral (Figure 3D) penumbra regions were evident in representative pictures as areas where HSP70 stained cells occur. In both Figure 3C,D these cells were further magnified in the corresponding insert allowing to detect HSP70 stained neuronal shapes. It is quite peculiar the detail in the topography of area penumbra which does not appear to span right according to a Euclidean geometric shape but it is rather a strip (grossly speaking) which indeed extends quite stochastically at high magnification according to the variability of small vessels carrying blood supply within microscopic brain regions. In this context, one may appreciate for instance in Figure 3C how the HSP70 staining passes from a strip-like pattern in the dorsal picture to an oblique line which encompasses the cortical-callosal border as shown by a cluster of arrows in the figure. This is plausible considering the topography concerning the course of small blood vessels which comply with the matter consistency varying at the border of brain structure as evident at gross anatomy (in a short note, toning down too loud implications, we may suggest to apply the chaos/fractal theory instead of Euclidean geometry to describe the shape of the area penumbra, when proceeding at the highest magnification in the brain matter).

### 2.4. From Light to Electron Microscopy through Identifying the Same Neurons Evident at Light Microscopy Semi-Thin Slices at TEM Electron Density

**Figure 4:**
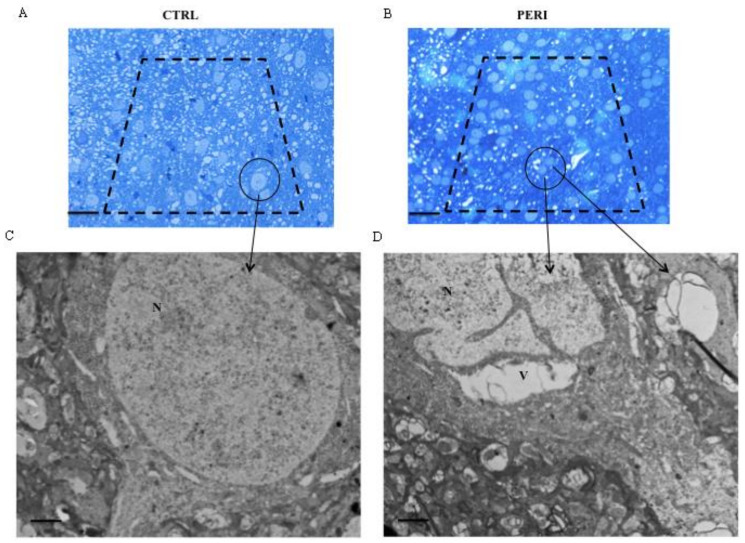
Representative pictures of contralateral cortex and penumbra area from an ischemic mouse. Semi-thin sections stained with toluidine blue from control cortex (**A**) and the penumbra cortical area (**B**) from an ischemic mouse. The dashed line indicates the area used for ultrathin section. In both representative semi-thin sections, the circle surrounds the neurons analyzed at TEM and reported in the corresponding panels (**C**,**D**). Scale bar = 60 μm (**A**,**B**), 0.3 μm (**C**,**D**). N = nucleus, V = vacuole.

In the process to detect the ultrastructure just in the very same area defined at light microscopy as area penumbra we used semi-thin sections from control (Figure 4A) and peri-infarct region of ischemic tissue (Figure 4B). Semi-thin slices, from 700 nm to 800 nm thick, stained with toluidine blue allow to identify the subcellular material further visualized in detail at ultra-thin (from 90 nm to 100 nm) thick TEM slices from control (Figure 4C) and peri-infarct region (Figure 4D).

As shown in Figure 4C neurons from controls are well shaped including their nucleus, while in peri-infarct area alterations are evidenced representatively as two big vacuoles witnessing for an ongoing cytopathology in the peri-infarct region (Figure 4D). In keeping with the scenario provided by the semi-thin slices, a marked homogeneity in pale basophilic material is abundant in the peri-infarct region (Figure 4D) compared with a high variability detected in the semi-thin slice from control (Figure 4C). These roundish pale bodies witness for a homogeneous emptying of cytosolic and nuclear structures as directly visualized in the ultra-thin slices at TEM. This preliminary step allows to proceed in the depth in the biochemical ultrastructure of the area penumbra to detect stoichiometry of HSP70 compartmentalization.

### 2.5. Ultrastructural Vacuolar Compartmentalization of HSP70

**Figure 5:**
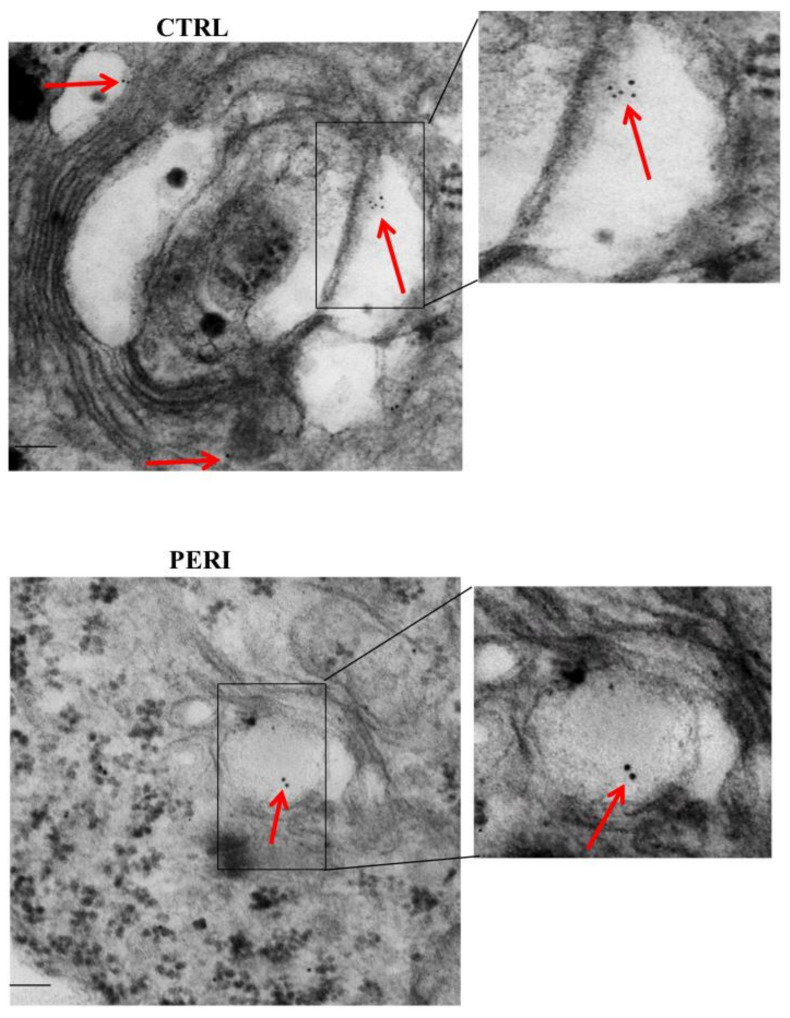
Representative polarization for HSP70. Representative TEM micrograph showing HSP70-positive vacuoles from the control cortex (**CTRL**) and the penumbra cortical area (**PERI**). Arrows point to HSP70 immuno-gold particles within vacuoles. The insert highlights the immuno-gold particles within vacuoles. Scale bar = 0.2 μm (low magnification), 0.1 μm (**inserts**).

**Figure 6:**
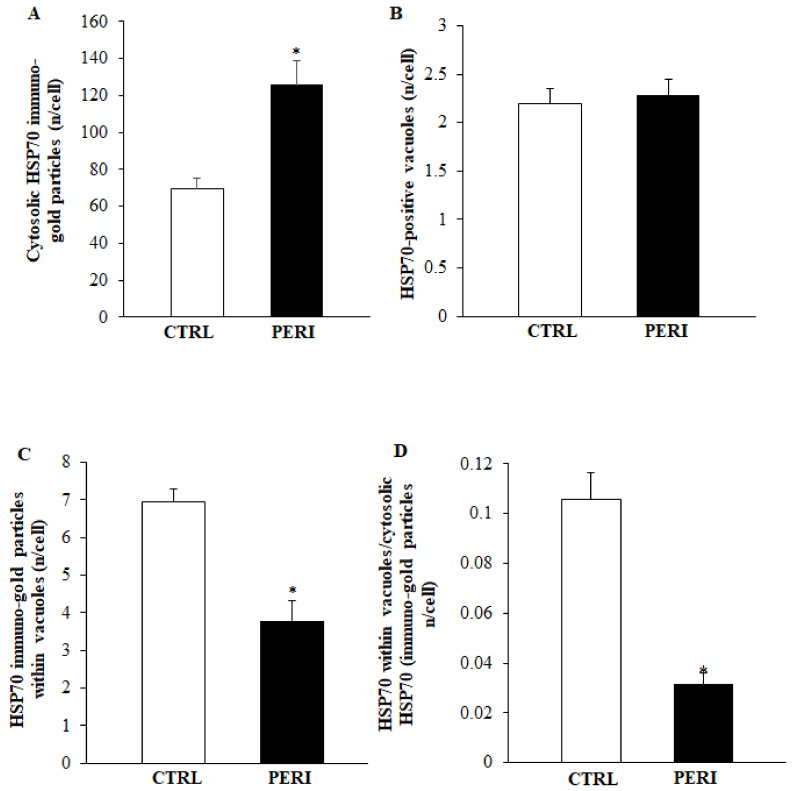
Representative polarization graphs for HSP70 in the dorsal cortex. Graphs report the cytosolic amount of HSP70 (**A**), the HSP70-positive vacuoles (**B**), the HSP70 immuno-gold particles within vacuoles (**C**), and the ratio of vacuolar to cytosolic HSP70 immuno-gold particles (**D**). Dorsal placement of area penumbra was dissected in the ipsilateral hemi-encephalon of ischemic mice (*n* = 3); analogous area was analyzed from the contralateral side. * *p* ≤ 0.05 compared with the contralateral dorsal cortex.

**Figure 7:**
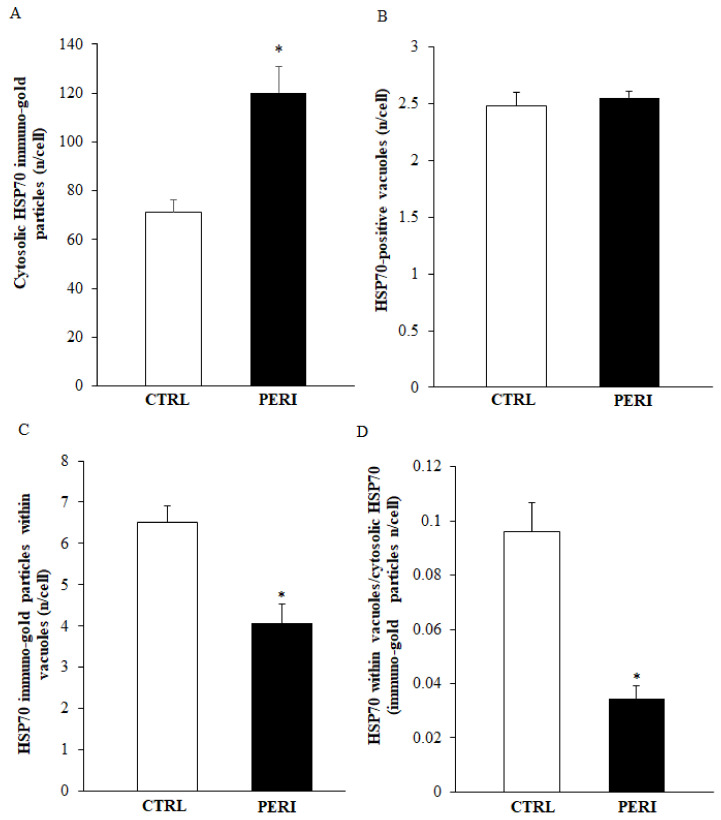
Representative polarization graphs for HSP70 in the ventral cortex. Graphs report the cytosolic amount of HSP70 (**A**), the HSP70-positive vacuoles (**B**), the HSP70 immuno-gold particles within vacuoles (**C**), and the ratio of vacuolar to cytosolic HSP70 immuno-gold particles (**D**). Ventral placement of area penumbra was dissected in the ipsilateral hemi-encephalon of ischemic mice (*n* = 3); analogous area was analyzed from the contralateral side. * *p* ≤ 0.05 compared with the contralateral ventral cortex.

As shown in the representative pictures of Figure 5, HSP70 immuno-gold particles were found to be abundant within autophagy-like vacuoles within the area penumbra of both control and peri-infarct neurons. In detail, clusters of HSP70 immuno-gold were found within double and multiple membranes delimited vacuoles. These clusters apparently were more abundant within the vacuoles of control neurons. When counts at ultrastructural morphometry were carried out from either the dorsal (Figure 6) or the ventral (Figure 7) peri-infarct region, the vacuolar compartmentalization of HSP70 could be assessed quantitatively. In detail, both in the dorsal and ventral region the occurrence of ischemia increased significantly the total amount of immuno-gold particles within the cytosol (graphs of Figure 6A and graph of Figure 7A). Despite the number of vacuoles was unmodified during ischemia (graphs of Figure 6B and graph of Figure 7B), the vacuolar compartmentalization of immuno-gold particles within vacuoles was suppressed two-fold with area penumbra compared with controls (graphs of Figure 6C and graph of Figure 7C). Most remarkable was the change in the ratio between vacuolar vs. cytosolic immuno-gold particles which was definitely suppressed within area penumbra (graphs of Figure 6D and graph of Figure 7D). This demonstrates that, within area penumbra HSP70, despite increased two-fold in the cell is dissipated from a vacuolar to a stochastic compartmentalization.

### 2.6. Ultrastructural of Mitochondrial Compartmentalization of HSP70

**Figure 8:**
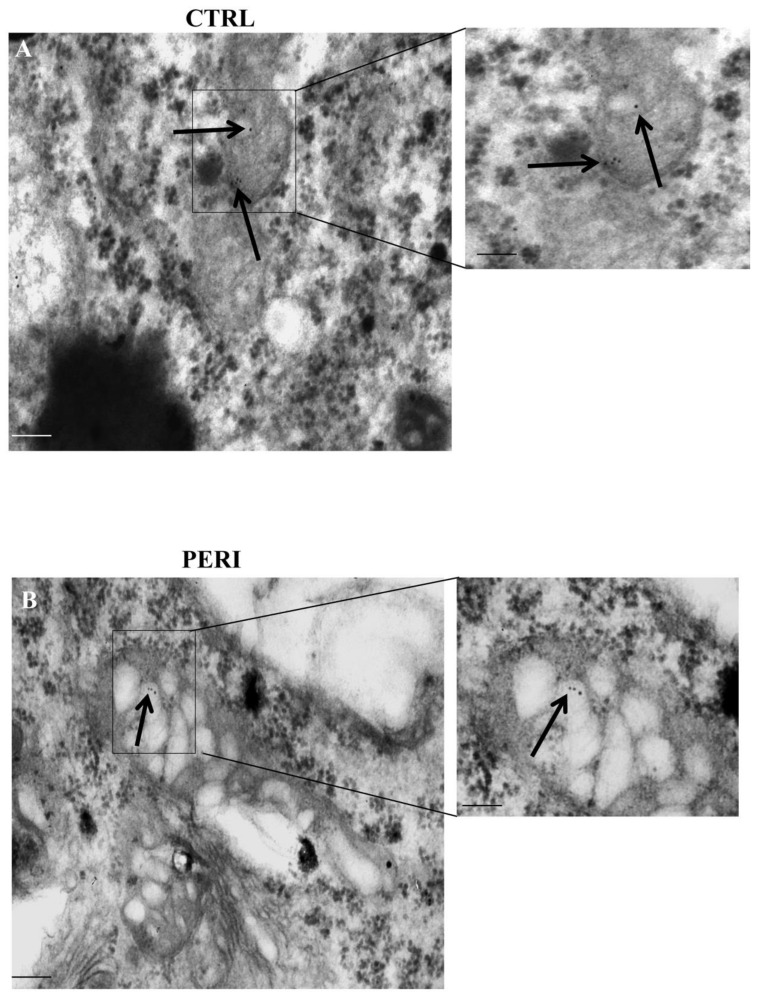
Representative polarization for HSP70 within mitochondria. Representative TEM micrograph showing a HSP70-positive mitochondrion from the control cortex (**CTRL**) and the penumbra cortical area (**PERI**). Arrows point to HSP70 immuno-gold particles within mitochondria. The insert highlights the immuno-gold particles within mitochondria. Scale bar = 0.2 μm (low magnification), 0.1 μm (**inserts**).

**Figure 9:**
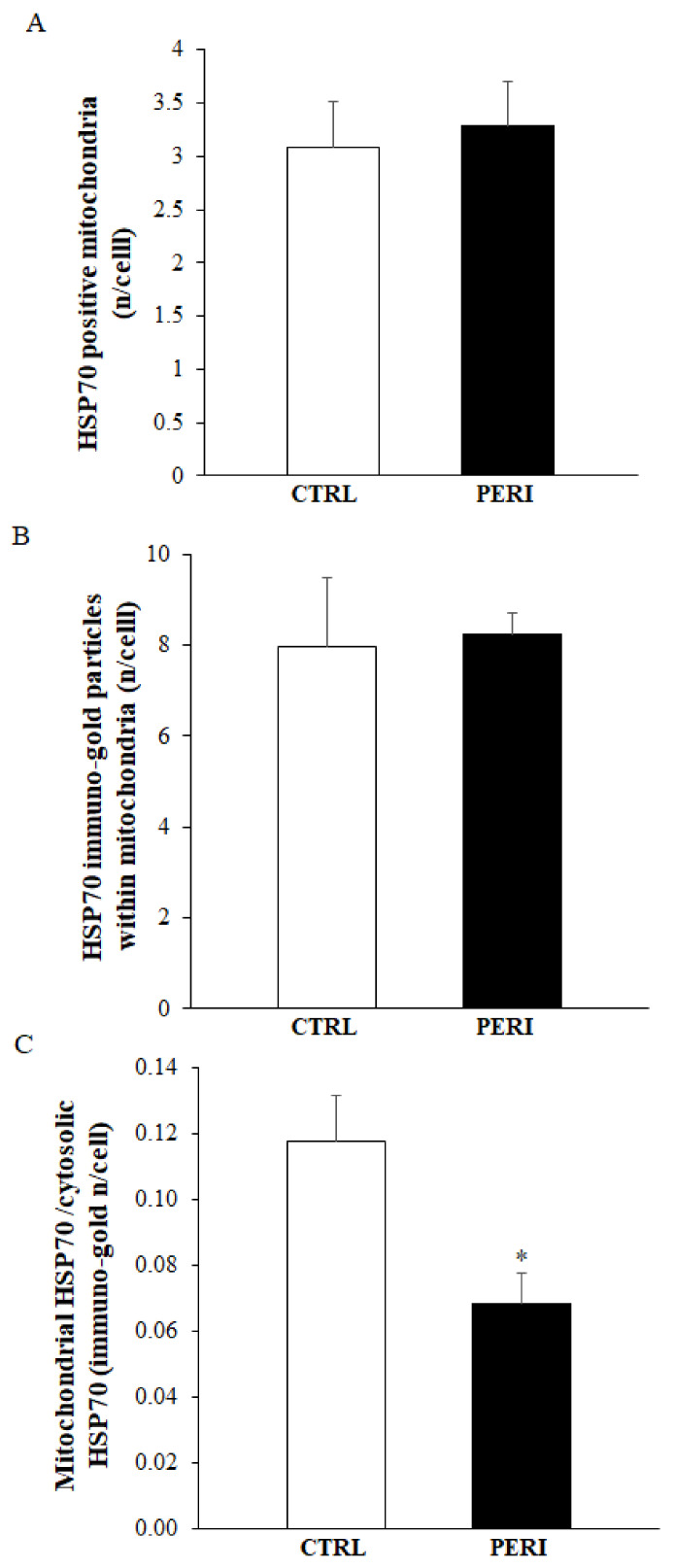
Polarization graphs for HSP70 within mitochondria in the dorsal cerebral cortex. Graphs report the HSP70-positive mitochondria (**A**), the HSP70 immuno-gold particles within mitochondria (**B**) and the ratio of mitochondrial to cytosolic HSP70 immuno-gold particles (**C**). Dorsal placement of area penumbra was dissected in the ipsilateral hemi-encephalon of ischemic mice (*n* = 3); analogous area was analyzed from the contralateral side. * *p* ≤ 0.05 compared with the contralateral dorsal cerebral cortex.

**Figure 10:**
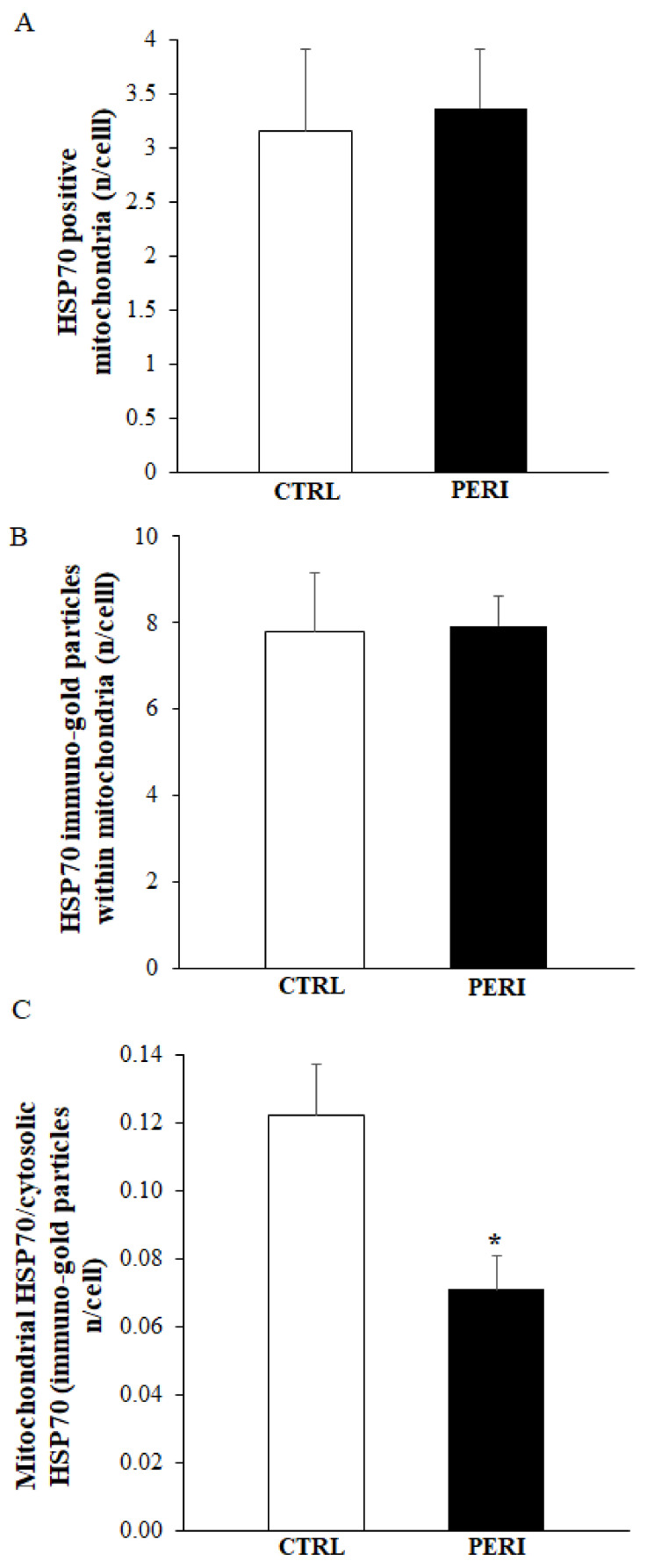
Polarization graphs for HSP70 within mitochondria in the ventral cerebral cortex. Graphs report the HSP70-positive mitochondria (**A**), the HSP70 immuno-gold particles within mitochondria (**B**) and the ratio of mitochondrial to cytosolic HSP70 immuno-gold particles (**C**). * *p* ≤ 0.05 compared with the control cortex.

Similarly to HSP70 compartmentalization within vacuoles, within area penumbra a marked compartmentalization of HSP70 occurs within mitochondria of control tissue peri-infarct region as shown by representative clusters of immuno-gold, which were detected at mitochondrial level (representative pictures of Figure 8). The placement of immuno-gold particles within mitochondria was suppressed within area penumbra compared with control tissue both in the dorsal and ventral peri-infarct region (graphs of Figure 9 and graphs of Figure 10, respectively). Since there is a total increase of HSP70 in the whole cell within area penumbra reported in Figure 6A and Figure 7A, the persistency of an equal amount of immuno-gold within mitochondria witnesses for a lack of mitochondrial placement of newly expressed HSP70 protein.

### 2.7. Number and Diameter of Mitochondria, Cytosolic Vacuoles and Cells within Area Penumbra

As reported in Table 1, the number of vacuoles is slightly non-significantly decreased as well as the number of mitochondria within the area penumbra. On the other hand, the area of both these organelles significantly increases (Table 1). Thus, considering that the increase in the total area of both vacuoles and mitochondria is increased compared with total cell area, the loss of HSP70 from its preferred compartments, when calculated based on surface units is further magnified. The correction provided by the organelles number and area augment the phenomenon of compartmental loss of HSP70 within area penumbra.

## 3. Discussion

The marker routinely applied in translational studies aimed to analyze area penumbra in the maturation of brain ischemia is represented by the chaperone heat shock protein 70 (HSP70) [1]. This protein marks quite selectively those cells placed in the so-called peri-infarct region, which indicates a brain strip placed at the border, between a brain area which is frankly and irreversibly damaged by the vascular insult and the surrounding normal tissue [1]. The routine identification of this brain region is provided at light microscopy, mostly by immunohistochemistry, which marks an overexpression of HSP70 [3,11,12,13]. Most cells possess both a constitutive and/or inducible HSP70 all activated in the area penumbra [14,15,16,17,18,19,20]. Despite a number of studies applying sophisticated procedures aimed to dissect the significance and molecular effects of HSP70 in area penumbra, to our knowledge no study so far investigated the compartmentalization of HSP70 by carrying out TEM and immuno-gold stoichiometry detection of HSP70 within area penumbra. In fact, despite being often described as overexpressed compared with control tissue [13], no study so far addressed the ultrastructural placement of the molecule and its potential misplacement directly visualized at TEM. Therefore, in the present study, the amount, placement and trafficking of HSP70 during ischemia compared with control conditions was assessed. Ultrastructural analysis allows to dissect the cell compartment(s) where the protein is placed in baseline conditions and, where it moves when the cell is part of area penumbra. The immuno-gold further allows to quantify the stoichiometric amount of the protein and the change, which is produced by ischemia even concerning compartmentalization. The study was designed based on a classic model of brain ischemia by permanent occlusion of the distal part of middle cerebral artery (MCA), where it is also known as the “Sylvian artery”. In this model, area penumbra is marked under light microscopy with immuno-peroxidase as routinely carried out. In order to strengthen the present analysis, stoichiometry of HSP70 was counted within specific cell compartments and the authentic moves of the protein towards and from specific compartments were assessed. The present results are expected to shed new light on the modulation of cytotoxic and cytoprotective significance of HSP70 compartmentalization, and to plan a therapy, which may restore the baseline placement of HSP70, when this is needed to fully exert its protective efficacy.

This manuscript presents unprecedented evidence for the subcellular placement inferring on the functional significance of HSP70 within specific cytosolic compartments. In this way, the protein, which works as a key molecule to stain ischemic penumbra, also takes part to modulate the survival of peri-infarct-placed neurons.

From a preliminary screening, we could detect immuno-gold staining HSP70 particles to be more abundant at the level of vacuole resembling those pertaining to the autophagy machinery and within mitochondria. Thus, in baseline conditions HSP70 is polarized within vacuoles and mitochondria Therefore, we counted the stoichiometric amount of this specific placement of HSP70 compared with cytosol in control conditions and following brain ischemia. Surprisingly though, within area penumbra these compartments significantly lose HSP70. In detail, the protein is expressed in higher amount in the whole cell, although with a lower polarization. In this way, it looks like that, in the presence of an ischemic insult, total HSP70 increases, but this occurs along with a loss of compartmentalization. Thus, ischemia dissipates HSP70 in the cytosol.

In fact, the remarkable polarization counted in control conditions within vacuoles and mitochondria, is lost within area penumbra. This suggests that puncta showing HSP70 aggregates within area penumbra compared with control tissue rather reflect cytosolic dissipated clusters of HSP70 rather than an increased topographical specificity for high HSP70 levels. Despite both vacuoles and mitochondria are deprived of HSP70 particles within penumbra cells, the amount of dissipation is greater for the vacuolar compared with mitochondrial compartment. The significance of such an alteration remains to be determined and deserves further studies to detect the actual change in HSP70 activities which may derive from such a de-localization.

For instance, it was shown that HSP70 within mitochondria may work as a chaperonin to rescue the cell from damage induced by oxidative species and free radicals [21]. A number of pathways may change their activity and indeed this occurs within area penumbra. This is the case of the ambiguous effects of autophagy and proteasome status within area penumbra which might be implicated in the depolarization of HSP70.

HSP70 appears to be induced by a variety of stressful conditions beyond ischemia, when distinct HSP70 proteins with a slight difference in their molecular weight, are increased during stressful conditions. These proteins are produced by two separate genes named HSP70.1 and HSP70.3 which again, are both sensitive to stressful conditions [22]. These genes code for the two isoforms of HSP70 which almost overlaps in their structure differing only for two amino acids [23].

The identification of area penumbra following an ischemic insult is also marked by the presence of CD86 and LAMP-1, which co-localize with HSP70 within exosome vesicles. This placement immediately suggests that vesicular HSP70 may help clearing cell from misfolded proteins filling multi-vesicular bodies (MVBs) which may be beneficial. This evidence leaves unturned the stone concerning the specific role played by HSP70 in health and disease. Again, even when dealing specifically with pathological condition a definite role of HSP70 in worsening or rescuing the pathology remains controversial. As a classic chaperone, HSP70 may foster directly protein folding and it addresses misfolded proteins to degradation through ubiquitination. In detail, HSP70 interacts directly with α-synuclein which is supposed to prevent from its toxicity. The interaction between HSP70 and α-synuclein is based on the flexible structure of HSP70 itself, which upon specific conformations increases its affinity for α-synuclein itself. Such a conformational plasticity and affinity shift is mostly mediated by a methyl transferase, which adds three methyl groups to a lysine residue of HSP70 [24]. By fostering the interaction with specific proteins HSP70 may either protect against protein toxicity by removing or extruding the protein from the cell as well as worsening the outcome of the pathological process fostering the spreading of disease-related proteins as shown by an excess of HSP70 which promotes tumor invasion [25]. This dual role is typical for chaperons and it is well represented by their ancestor PrPc. The chaperone activity of HSP70 was studied in detail at molecular level. In fact, HSP70 forms a surface which packs misfolded ubiquitinated substrates, where on the other side, an analogous surface intercept co-chaperone (CHIP, HSP90) [26]. Thus, while HSP 70 is properly defined as a chaperone, other heat shock proteins, such as HSP40 and HSP90 are co-chaperones. Of these, α-synuclein is an important co-chaperone which may interact with HSP70. The substrate ubiquitination depends on the interaction between the E3 enzyme (ubiquitin-ligases among, which parkin) with the co-chaperone such as CHIP (HSP90). The time sequence would be the following: the chaperone (HSP70) binds the misfolded substrate, in this conformation the co-chaperone packs the complex and serves as the anchor where ubiquitin ligase binds to catalyze the ubiquitination of the packed substrate itself. All these activities are promoted when HSP70 is placed within vacuoles. Thus, they may be lost following HSP70 dissipation.

In conclusion, the present manuscript provides the identification and quantitative stoichiometry and placement of the chaperone protein HSP70 within area penumbra compared with control neurons. Despite, the staining of area penumbra is carried out routinely to mark area penumbra, no study so far investigated the cell compartments where HSP70 is placed. Similarly, no investigation considered the potential misplacement of HSP70 within area penumbra compared with control tissue. The net effect of HSP70 provides neuroprotection within area penumbra neurons, thus an increase in the protein appears as a compensatory attempt to counteract a potential brain damage. In fact, mice knocked out for HSP70 possess a wider infarct volume compared with wild types. Nonetheless, the partial energy depletion which characterizes neurons placed within area penumbra does not allow to exploit at best HSP70 overexpression. Thus, it is likely that the significance of the shift in the polarization of HSP70 within area penumbra from mitochondria and vacuoles towards non-compartmentalized cytosol remains uncertain. Assuming a strategical placement of HSP70 within these compartments in control tissue, it is likely that a loss of such a compartmentalization is rather detrimental than compensatory. In fact, it seems to be produced by an energy failure, which otherwise counteracts entropy opposing compartmentalization of functionally active molecules. HSP70 is a chaperone protein, which works effectively both at mitochondrial level and within vacuole compartments mostly of autophagy nature. Thus, the outcome to improve the fate of neurons place within area penumbra would be to administer a drug, which reverts such a potential deleterious shift to prevent HSP70 dissipation.

## 4. Materials and Methods

### 4.1. Permanent Focal Ischemia in Mice

Ten adult C57BL/6J male mice (8 weeks old, in-house colony, Neuromed, Pozzili, Italy) weighing 25 g, were housed under controlled conditions (ambient temperature, 22 °C; humidity, 40%) on a 12 h light-dark cycle with food and water *ad libitum*. The experimental protocol was approved by the Ethical Committee of Neuromed Institute (Pozzilli, Italy) further supervised by the Italian Ministry of Health. The animals were anaesthetised with chloral idrate (400 mg/kg, i.p.). With the aid of an operating stereomicroscope, an incision was made between the outer canthus of the eye and the external auditory meatus. The temporal muscle was bisected and retracted to expose the temporo-lateral surface of the skull. The MCA was exposed by small craniotomy (0.5 mm), which was carried out by using a surgical drill. A thin layer of the drilled bone was preserved to protect the dura mater and cortex surface from mechanical damage and thermal injury, then, this remaining bone was manually removed to reach out the distal course of the middle cerebral artery to coagulate the vessel. The complete and permanent interruption of the blood flow was visualized by stereo-microscope. The incisions of the temporal muscle and overlying skin were sutured by using 5/0 polyglactin sutures [27,28]. All the procedure was carried out by keeping body temperature was close to 37 °C. MCA occlusion was performed on 7 animals; four animals were dedicated to histologic assessment and count of the infarct volume, including representative pictures to assess by light microscopy HSP70 staining and area penumbra placement. Three additional mice were processed for transmission electron microscopy by using specimen from dorsal and ventral area penumbra (as detected at light microscopy and further oriented by toluidine bleu in preparatory semi-thin section. This allowed specific tissue sectioning of area penumbra from the ischemic side and equivalent regions from the contralateral side. Sham-operated animals (*n* = 3) undergo the same surgical procedure, but the final step of middle cerebral artery cauterization. Animals were killed at 24 h after MCA occlusion.

### 4.2. Histology

Brains were dissected out and immediately placed into a Carnoy fixing solution composed of ethyl alcohol (60%), acetic acid (10%), and chloroform (30%). Twenty hours later, brains were placed into 70% ethanol until they were included in paraffin. The paraffin included brains were cut at a microtome (RM2125, Leica Microsystem, Milan, Italy) into 10 μm-thick coronal slices, which were regularly spaced every 550 μm through the extension of the ischemic region (Figure 1). Sections were dewaxed and processed for thionin staining (a type of Nissl staining) for histologic assessment of neuronal degeneration. Based on this topography we punched out specimen for semi-thin sections in order to select the specific area to be analyzed under quantitative morphometry using immuno-gold transmission electron-microscopy to quantify the subcellular placement and trafficking of HSP70 (Figure 2).

### 4.3. Immunohistochemistry

De-waxed brain slices were treated for antigen retrieval through a citrate buffer solution (pH 6.0, for 10 min) in heating up to boiling conditions. Slices were incubated with 0.1% Triton solution (Sigma Aldrich, St. Louis, MO, USA) for 15 min and were soaked in 3% hydrogen peroxide to block endogenous peroxidase activity; then they were incubated overnight at +4 °C with mouse anti-HSP70 (1:100; R&D Systems, Minneapolis, MN, USA; Antibody registry: AB_2119388); these slices were further incubated with anti-mouse biotinylated secondary antibodies (1:200; Vector Laboratories, Burlingame, CA, USA) for 1 h at room temperature. For detection 3,3-diaminobenzidine tetrachloride (Sigma Aldrich, St. Louis, MO, USA) was used. In some cases slices were counterstained with Mayer’s haematoxylin (Diapath, Martinengo, BG, Italy) for 6 min. Negative experimental control slices were similarly process but omitting the primary antibody. This antibody detects the inducible form of HSP70 which is visible as a strip of stained tissue (Figure 3).

### 4.4. HSP70 Detection

Considering that various antibodies are used routinely to detect HSP70 to mark area penumbra, in the present study, when performing transmission electron microscopy we used an anti-HSP70 antibody directed against all various isoforms of HSP70. This allows to detect all shifting in subcellular compartmentalization of the protein. This antibody was revealed by immuno-gold particles, thus providing the first ultrastructural evidence of HSP70 and its misplacement within area penumbra. Such an antibody stains a panel covering all various isoforms of HSP70 along which undergo over-expression within area penumbra. This antibody was added to the specific antibody, which marks the isoform HSP70/HSPA1A (Figure 3), which is inducible under a variety of stressful conditions where HSP70 is expected to provide neuroprotection and frankly behaves as a chaperon. In this way, since both specific and non-specific primary antibodies are routinely used in the analysis of area penumbra it was critical in these settings to refine the protein detection with two types of primary antibody to cast here unbiased evidence concerning the subcellular compartmentalization of the protein. This was done to check the consistency of the subcellular compartmentalization data independently by the specificity of HSP70 isoform being detected. Experiments to detect HSP70 were carried out at 24 h after MCA occlusion. This time interval was selected based on two main point. Although early pathological findings of infarction develop already at 1 h after MCA occlusion, 24 h are required to fully mature the infarct area [29]. The time course of the protein pattern expressed within area penumbra demonstrates that HSP70 began to be induced during the first few hours after tMCAO, it peaks at 24 h, and it decreases within 48 h. Thus 24 h corresponds to the time interval when inducible HSP70 peaks.

### 4.5. Transmission Electron Microscopy (TEM)

Mice were perfused with fixing solution (paraformaldehyde 2.0% and glutaraldehyde 0.1% in 0.1 M PBS pH 7.4). Blocks of cerebral cortex were dissected based on Figure 2 both in the dorsal and ventral placement of area penumbra in the ipsilateral hemi-encephalon of ischemic mice and analogous areas were dissected from the contralateral side (*n* = 3). In detail, by using a matrix we cut a 1 mm slice region along the rostro-caudal axis to obtain 1 mm thick coronal slice each one further punched to dissect squared slices measuring 1 mm × 1.3 mm placed at the dorsal and ventral border of the ischemic region. Dissected specimens were immersed in the perfusing solution overnight at 4 °C. After washing in PBS (0.1 M), samples were post-fixed in 1% osmium tetroxide (OsO_4_) for 1 h at 4 °C. The same procedure was carried out in the contralateral (non-ischemic) hemisphere. The specimens were dehydrated in a gradient of ethanol solutions and finally embedded in epoxy resin. The concentration of the fixing and post-fixing solution along with the use of the epoxy embedding resin were validated in our previous studies regarding the immuno-gold-based ultrastructural morphometry [30]. In fact, the combination of aldehydes, OsO_4_, and epoxy resin allows a minimal epitope covering, while preserving subcellular architecture [31,32,33]. For identification of the peri-ischemic and contralateral area, 1–2 μm thick serial sections, obtained with a porter Blum MT-1 or an ultramicrotome Reichert-Jung, were stained with 1% toluidine blue and 1% methylene blue in 1% sodium tetraborate and observed with at light microscopy (semi-thin sections, Figure 4). From these sections the isosceles trapezoid area was selected on the embedded tissue blocks to be further cut at ultramicrotome (Leica Microsystems, Wetzlar, Germany) to proceed with ultrastructural analysis.

Either plain transmission electron microscopy or post-embedding immuno-electron microscopy were carried out on ultrathin sections cut at ultramicrotome, and after staining with uranyl acetate and lead citrate, and they observed using a JEM SX100 electron-microscope (JEOL, Tokyo, Japan) at an acceleration voltage of 80 kV.

### 4.6. Post-Embedding Immuno-Electron Microscopy

The immuno-electron microscopy technique allows morphometric analysis and detection of proteins placement in different cell compartments, such as vacuoles. In fact, the high resolution of gold-conjugated particles allows the detection and localization of the stoichiometric antigen-antibody reaction in subcellular structures. Ultrathin sections were incubated on droplets of aqueous sodium metaperiodate (NaIO_4_) for 30 min, at 21 °C to remove OsO_4_ as much as needed to unmask antigens [34]. The sodium metaperiodate attacks the hydrophobic alkane side-chains of epoxy resin thus making sections more hydrophilic, which allows an intimate contact between immuno-gold-conjugated antibodies and section surface antigens [35]. Nickel grids were incubated with aqueous saturated NaIO4 solution for 30 min, at 21 °C. After washing in PBS grids were incubated in drops of blocking solution (10% goat serum and 0.2% saponin in PBS) for 20 min at 21 °C. Then grids were further incubated in a humidified chamber overnight at 4 °C with primary antibody anti mouse-HSP70 (ab5439, Abcam, Cambridge, UK, diluted 1:20) in ice-cold solution in PBS containing 1% goat serum and 0.2% saponin. After washing in cold PBS, ultrathin sections were incubated in the gold-conjugated secondary antibodies (10 nm gold particles, BB International, Treviso, Italy), diluted (1:20) in blocking buffer (1% goat serum and 0.2% saponin in PBS) for 1 h at 21 °C.

After rinsing in PBS, grids were incubated with 1% glutaraldehyde for 3 min, they were washed in distilled water to remove salt traces and prevent uranyl acetate precipitation; grids were counterstained with a saturated solution in distilled water of uranyl acetate and lead citrate to be ready for electron microscopy analysis. Sections for methods control were incubated with secondary antibody only.

### 4.7. Ultrastructural Morphometry

Counts of immuno-gold particles (10 nm) were performed at TEM at the minimal magnification (8000×) in which both immuno-gold particles and cell organelles can be identified [36]. To count the immuno-gold particles in cortical neurons we started observing a grid square corner in order to scan the whole section within that grid square, which was randomly identified. Assessments of vacuoles and measurement of immuno-gold particles were carried out according to Lenzi et al. [33]. We scored the vacuoles as single, double or multiple membranes containing cytoplasmic material and electron-dense structures according to previous studies [37,38]. In each neuron, we counted HSP70 immuno-gold particles as follows: (i) the total number of immuno-gold placed either in the total cytoplasmic areas; (ii) the number of wide sparse, not compartmentalized, immuno-gold particles within the cytosol; (iii) the number of HSP70 within vacuoles; (iv) the number of HSP70 within mitochondria.

### 4.8. Statistics

For ultrastructural morphometry, values were expressed as the mean + SEM of the following measurements: (i) number of HSP70 per cell; (ii) number of cytosolic HSP70 per cell; (iii) number of HSP70 positive vacuoles per cell; (iii) number of HSP70-immuno-gold within vacuoles per cell; (iv) number of HSP70 positive mitochondria per cell; (v) number of HSP70-immuno-gold within mitochondria per cell. Moreover, values for ultrastructural morphometry were also expressed as a ratio of the number of HSP70 immuno-gold particles within vacuoles out of the total number of HSP70 immuno-gold particles, which were counted in the cytosol, as well as (ii) the number of HSP70 immuno-gold particles within mitochondria out of the number of cytosol HSP70 immuno-gold particles, which were counted in the cytosol. Values were reported as the mean or the mean percentage ± S.E.M. per cell from a total of 60 cells for each tissue block. Inferential statistics to compare groups was carried out by using one-way analysis of variance (ANOVA), with Scheffè’s post-hoc analysis (H_0_, null hypothesis, was rejected when *p* ≤ 0.05).

## Figures and Tables

**Table 1:** Ultrastructural morphometry of HSP70 within whole cell compartments.

	Control Dors/Ventr ^1^	Ischemia Dors/Ventr ^1^
Number of mitochondria/cell	20.77 ± 1.49/22.52 ± 1.37	19.82 ± 1.82/19.7 ± 0.62
Number of vacuoles/cell	6.97 ± 0.19/6.9 ± 0.39	6.58 ± 0.8/6.05 ± 0.87
Mean of mitochondria area	0.25 ± 0.01/0.24 ± 0.002	0.35 ± 0.003/0.36 ± 0.008
Mean of vacuoles area	0.70 ± 0.02/0.67 ± 0.02	2.06 ± 0.10/2.021 ± 0.06
Total mitochondrial area/cell	5.19 ± 0.3/5.46 ± 0.36	6.71 ± 0.10/7.57 ± 0.06
Total vacuolar area/cell	4.79 ± 0.36/4.79 ± 0.38	12.93 ± 2.08/14.59 ± 1.58

^1^ Dorsal and ventral placement of area penumbra were dissected in the ipsilateral hemi-encephalon of ischemic mice (*n* = 3); analogous areas were analyzed from the contralateral side.

## Data Availability

The data that supports the findings of this study are available from the corresponding author upon reasonable request.
